# Community-level externalities in child health: Evidence from Sub-Saharan Africa

**DOI:** 10.1371/journal.pone.0341218

**Published:** 2026-01-21

**Authors:** Emmanuel Skoufias, Katja Vinha

**Affiliations:** 1 Lee Kuan Yew School of Public Policy, National University of Singapore, Singapore; 2 Independent Consultant, Nashville, Tennessee, United States of America; Public Library of Science, UNITED KINGDOM OF GREAT BRITAIN AND NORTHERN IRELAND

## Abstract

We use data from twenty countries from the harmonized versions of the Demographic and Health Surveys (DHS) for countries in Sub-Saharan Africa to investigate the extent to which contextual factors such as the level of women’s schooling in the community and the fraction of households in the community with improved water and improved sanitation facilities have an impact on child health in the short-term and in the long-term. Short-term child health is measured by the incidence of reported fever and diarrhea in the two weeks preceding the survey, and long-term child health is measured by child height for age z-scores. We employ the hybrid modeling approach that combines the strengths of random-and fixed-effects models, and considers the role of unobserved heterogeneity at the community level, so far ignored in the literature, to account for all the potential synergies in externalities at the community level. We test the sensitivity of the estimates of externalities to alternative specifications for cluster-level unobserved heterogeneity. The empirical analysis confirms the role of externalities at the community level and shows that the size of these externalities varies between rural and urban areas and by outcome considered. Our results reaffirm the paramount role of externalities in maternal education on child height-for-age and on the incidence of fever and diarrhea in both urban and rural areas. No significant externalities are associated with the fraction of households in the community having improved water, though the fraction of households in the cluster with improved toilets are shown to be significantly associated with a lower probability of fever and diarrhea and improved child height-for-age, with some of these effects varying between urban and rural areas.

## Introduction

The positive contribution of maternal education and access to improved water and sanitation facilities on the nutritional status of children is well established in the literature. Numerous studies based on observational data have shown that maternal education, water quality, sanitation, and handwashing (WASH) in a household are strongly associated with linear growth of children living in the same household [1–6]. Understandably, maternal education and WASH constitute essential components of the underlying causes and determinants of child undernutrition in the UNICEF conceptual framework [7].

However, the question of whether the investments of any household in the community have broader social and economic impacts that extend beyond the health and nutritional impacts on household’s own children has received rather uneven attention in literature. Much of the focus has been on community level externalities associated with maternal education [3, 8–14]. Mothers with less or no education, for example, appear to be able to benefit from information and advice provided, and even the cultural attitudes exhibited by more educated women in the community. Thus, the impacts from a mother’s education diffuse across neighbors.

Such externalities and contextual effects may also be at work with individual household investments in water and sanitation. Household investments in sanitation can have an impact across the wider neighborhood. Such investments may reduce the proliferation of and exposure to pathogens in the neighborhood’s air and soil and thus indirectly contribute to the nutritional and health status of children in neighboring households who may not have proper sanitation facilities. Individual investments improving access to water, in terms of water quality, water quantity, and/or water collection times by any given household may also result in improved overall community child nutrition. If water that is cleaner or more available increases the frequency of food preparation then pathogens may have less time to multiply sufficiently to cause disease. However, the extent to which individual investments in improved water impact the nutritional status of neighborhood children without improved water facilities needs empirical validation.

The extant empirical evidence on the externalities across households from investments in improved basic services at the neighborhood level is rather scarce. Studies generally focus on the potential spillover effects of one policy variable of interest, such as maternal education or sanitation, at a time and ignoring any potential interlinking and synergies that may arise at the community level. For example, Andres et al. [15] and Larsen et al. [16] focus on estimating potential externalities in community level sanitation without considering the externalities that might be present with higher maternal education or higher incidence of uncontaminated water at the community level. This practice of focusing on only one variable at a time may exaggerate the externalities from improved sanitation, because it attributes to sanitation, incorrectly, externalities that might be due to synergies with other community-level characteristics. For example, in communities with better sanitation, a higher fraction of mothers may have completed primary schooling, or a higher fraction of households may have access to improved water. Educated mothers are more likely to utilize sanitation infrastructure effectively and teach adequate hygienic practices and thus it is not only the improved sanitation infrastructure but also the better educated population that in fact improves the outcomes of interest. To the extent that maternal education, improved sanitation and improved water services at the community level are correlated, the omission of any of these factors is likely to result in biased estimates of the externalities under consideration due to omitted variable bias. To our knowledge the study by Alderman et al. [8] is the only exception that investigates community level externalities in maternal education, sanitation and water services at the same time.

Our study focuses on Sub-Saharan Africa (SSA). It employs data from twenty countries from the harmonized versions of the Demographic and Health Surveys (DHS) to investigate the extent to which contextual community factors impact child health and nutrition. Specifically, we estimate the impacts of women’s schooling, access to improved water and access to improved sanitation facilities at the community level on child health in the short-term and in the long-term. Short-term child health is measured by the incidence of reported fever and diarrhea in the two weeks preceding the survey, and long-term child health is measured by child height for age z-scores We use DHS clusters to define community level measures.

Inferences regarding externalities at the community level are by necessity based on estimates of the coefficients of community-level variables such as the fraction of females in the cluster with a certain level of education or the proportion of households with improved sanitation and/or improved water facilities. Random-effects (or two-level hierarchical) models, that decompose additively the regression error term into a community-wide component and a household-specific or idiosyncratic component, allow researchers to estimate the effects of community-level variables of interest on child nutrition. Inferences, however, can be unbiased only under the questionable assumption that the community level random-effects (community-level unobserved factors) are uncorrelated with the observed household and observed community-level covariates. For example, the quality of schools in the community is not observable, but may be correlated with the educational attainment variables of mothers and the community. In contrast, the within-community or within-cluster (W) or fixed -effects method that is typically the preferred method for eliminating unobserved heterogeneity at the community level, does not allow estimation of the effect of community-level variables on child nutrition. This is because the within community method models out variance across communities and makes any correlations between any unobserved heterogeneity at the community-level and covariates irrelevant.

This paper contributes to the topic of community level externalities and investments in human capital and basic services in two ways. First, the analysis pays close attention to the potential role of unobserved heterogeneity at the community level by employing an estimation strategy that addresses this problem directly. The available estimates of externalities in the literature are based on the OLS method that essentially assumes on an ad-hoc basis that there are no unobserved community factors in the regression residual that may be correlated with the included regressors. The strategy adopted here separates the effects of variables into two associations, one at the child level or within-community effects and one at the community level or between-community effects and thus combines the strengths of random- and fixed-effects models [17–19]. Notable examples of this estimation strategy are the hybrid model [20] and the correlated random-effects (CRE) model [21–23]. Such models yield fixed-effects (or within-cluster) estimates of the child or household level (level one) covariates that are unbiased by community level unobserved heterogeneity, while at the same time yielding unbiased estimates of the effects of community-level (level two) covariates and thus more reliable estimates of the community-level externalities on child nutrition. The hybrid/CRE model also allows consideration of all the potential synergies in externalities that might influence child nutrition and the incidence of illness through the interactions of community-level maternal education, improved sanitation and water services. Second, the method employed offers the opportunity to investigate the sensitivity of the estimates of externality effects to alternative specifications of the community/cluster-level unobserved heterogeneity. Different specifications of the relationship between cluster-level unobservables and the cluster mean values of some or all level one variables can shed light on the extent to which the estimates of externality effects are robust.

Empirical evidence on the significant role of externalities among households informs public policy decisions on the amounts of public investments in education and basic water and sanitation infrastructure, the extent to which the costs of such investments need to be subsidized so that more households can afford the service, and whether public resources should be targeted to the community as opposed to the household. Empirical evidence can also shed light on the question of whether these public investments have the potential of acting as substitutes for or complements to the private investments that individual households may be able (or unable) to undertake towards improving child health and nutrition. When there are significant positive externalities in maternal education, there is more scope for public investment as the benefits not only accrue to the mother’s own children but also other children in her community.

## Materials and methods

The analysis is based on data from 20 countries in SSA for children aged zero to 59 months of age. The data were extracted from the harmonized versions of DHS surveys. This study analyzed secondary data that are publicly available and which fully de-identified. We did not have access to information that could identify individual participants during or after data collection. The research did not involve direct interaction with human participants or animals and the use of these data does not constitute human subjects research. The complete data for all the countries analyzed in our paper, are publicly accessible through the IPUMS-DHS website https://www.idhsdata.org/idhs/ with prior registration with DHS [24]. The data were first accessed on April 2, 2021. Table 1 below lists the countries covered and the years of the surveys used in the analysis.

**Table 1:** List of harmonized DHS surveys (IPUMS) analyzed.

County	Years
Angola	2015
Benin	2001, 2011, 2017
Burkina Faso	2003, 2010
Cameroon	2004, 2011
Chad	2014
Cote d’Ivoire	2011
Ghana	2003, 2008, 2014
Guinea	2005, 2012, 2018
Lesotho	2004, 2009, 2014
Liberia	2006, 2013
Madagascar	2008
Malawi	2010, 2016
Mali	2001, 2006, 2018
Mozambique	2011
Namibia	2000, 2006, 2013
Nigeria	2003, 2008, 2013, 2018
Senegal	2005, 2010−16
Tanzania	2010, 2015
Zimbabwe	2005, 2010, 2015
Zambia	2007, 2013, 2018

Notes: The data can be downloaded from **https://www.idhsdata.org/idhs/**.

The child health-related variables we analyze are the reported incidence of fever and diarrhea and child heigh-for-age. The incidence of child diarrhea and fever as reported by mothers, is collected by the DHS surveys for the 2 weeks prior to the date of the survey. Post-natal linear growth as measured by height-for-age (HAZ) compares the child’s height (or length if measured lying down) to the height (length) distribution of healthy children of the same age and sex, such that HAZ measures the child’s height in terms of standard deviations from the median height. Thus, a child whose height corresponds to the median height of healthy children of the same age and sex will have a HAZ of zero. A child taller than the “median” child will have a positive value and those shorter than the “median” child will have a negative HAZ value.

The three policy-related variables of interest are the years of education of the child’s mother, the household’s access to an improved water source and the household’s access to an improved and non-shared toilet facility. Both of the infrastructure variables are constructed as binary variables at the household level and defined based on WHO/UNICEF Joint Monitoring Program guidelines. We also control for other factors which consistently available for our set of surveys and are commonly included to explain child nutrition or health [6,8–14,16]. Additional variables used as controls include: the child’s age in months (7 categories), the child’s gender, whether the child is mother’s first child, whether mother’s height is more than 160 cm, whether the house has a high quality roof, total number of household members, total number of children under 5 in the household, travel time to urban center with at least 20,000 people, household asset index (5 quintiles), binary variables for year of survey, and binary variables for month of survey. Table 2 presents the mean and standard deviation of all the variables in the urban and rural samples used to analyze the incidence of fever in our analysis. The sample used for the analysis of child diarrhea is practically identical, while the sample used to analyze child HAZ is a smaller subset of this sample, due to missing or outlier observations on HAZ.

**Table 2:** Means (std. deviations) of variables in the urban and rural sample used to analyze the reported incidence of fever among children 0 to 60 months of age.

	Urban	Rural
Number of observations	80,856	183,086
Child HAZ based on WHO	−1.104 (1.635)	−1.518 (1.695)
fever	0.176 (0.381)	0.194 (0.396)
diarrhea	0.137 (0.344)	0.147 (0.354)
Mother’s years of education	6.064 (4.960)	3.097 (3.884)
Access to improved drinking water	0.754 (0.430)	0.460 (0.498)
Access to improved, non-shared sanitation facility	0.374 (0.484)	0.199 (0.399)
No access to improved water and improved toilet	0.174 (0.379)	0.456 (0.498)
With access to one of improved water or improved toilet	0.524 (0.499)	0.429 (0.495)
With access to both improved water and improved toilet	0.302 (0.459)	0.115 (0.319)
Child is girl	0.493 (0.500)	0.496 (0.500)
First born	0.247 (0.431)	0.188 (0.390)
Mother’s height is greater than 160 cm	0.347 (0.476)	0.306 (0.461)
Roof material of high quality	0.872 (0.334)	0.492 (0.500)
Total number of household members	7.410 (4.770)	8.002 (5.131)
Number of children under 5 in household	2.121 (1.271)	2.426 (1.538)
Child 0–6 months	0.131 (0.338)	0.132 (0.338)
Child 7–12 months	0.113 (0.317)	0.113 (0.316)
Child 13–18 months	0.110 (0.312)	0.112 (0.316)
Child 19–24 months	0.098 (0.297)	0.095 (0.293)
Child 25–36 months	0.194 (0.396)	0.191 (0.393)
Child 37–48 months	0.191 (0.393)	0.191 (0.393)
Child 49–60 months	0.163 (0.369)	0.165 (0.372)
Poorest wealth quintile	0.050 (0.217)	0.323 (0.468)
Second poorest wealth quintile	0.075 (0.264)	0.291 (0.454)
Third wealth quintile	0.171 (0.377)	0.220 (0.414)
Second highest wealth quintile	0.310 (0.463)	0.130 (0.337)
Top wealth quintile	0.394 (0.489)	0.036 (0.186)

**Source:** Author calculations based on DHS surveys listed in Table 1.

To date, the literature on externalities associated with maternal education and basic service coverage, has relied on the following stylized econometric model


$$Y_{ij}=\beta_{\text{0}}+\beta_{\text{1}\text{1}}X_{ij}^{\;\text{1}}+\;\beta_{\text{1}\text{2}}X_{ij}^{\text{\textbackslash{} 2}}+\delta Z_{j}+\varepsilon_{ij}$$ (1)

where $Y_{ij}$ is the outcome variable of interest such as the Height-for-Age z-score (HAZ) of child i in community j, $X_{ij}^{\text{\textbackslash{} 1}}$ denotes policy variables of interest, such as the level of a mother’s education, or whether the household has improved water or improved sanitation, and $X_{ij}^{\text{\textbackslash{} 2}}$ is other child and household control variables such as child age, and household size. The error term $\varepsilon_{ij}$ in the regression summarizes the influence of all other unobserved variables at the individual and at the community level on the outcome variable.

An implicit and untested assumption of the existing studies focusing on community level externalities is that the error term consists of two components


$$\varepsilon_{ij}=\;u_{j}\;+\;e_{ij}=\;\;\gamma {\overset{―}{X}}_{j}^{\text{1}}+\;e_{ij}$$ (2)

where $u_{j}$ is a community level error term that can be accounted for by including the community level mean value of the main policy variable of interest, i.e., $\gamma {\overset{―}{X}}_{j}^{\text{1}}$, such as the community average years of education of mothers, or the fraction of household in the community with improved water or toilets. Based on this specification, equation (3) below is estimated using OLS, i.e.,


$$Y_{ij}=\beta_{\text{0}}+\beta_{\text{1}\text{1}}X_{ij}^{\text{\textbackslash{} 1}}+\;\beta_{\text{1}\text{2}}X_{ij}^{\text{\textbackslash{} 2}}+\gamma {\overset{―}{X}}_{j}^{\text{1}}+\delta Z_{j}+e_{ij}$$ (3)

Community/cluster-level externalities are investigated by focusing on the sign and significance level of the estimate of the parameter γ, the coefficient of the community-level average of maternal years of schooling or the fraction of households in the community with improved water and/or improved toilet facilities. It is important to bear in mind that the standard practice is to exclude from the regression model the community level means of the other policy variables and child characteristics and household control variables in $X_{ij}^{\text{2}}$. The key assumption for any credible evidence on the significant role of externalities based on this approach is that $E\left(e_{ij}|X_{ij}^{\text{\textbackslash{} 1}},\;X_{ij}^{\text{\textbackslash{} 2}},{\overset{―}{X}}_{j}^{\text{ 1}},\;Z_{j}\right)=\text{0}$, which implies that there are no omitted community-level confounders, meaning that $\gamma {\overset{―}{X}}_{j}^{\text{1}}$ is a perfect proxy for all the community-level unobservables denoted by $u_{j}$ in equation (2) above. If any omitted confounders are correlated with the covariates, i.e., $E\left(e_{ij}|X_{ij}^{\text{\textbackslash{} 1}},\;X_{ij}^{\text{\textbackslash{} 2}},{\overset{―}{X}}_{j}^{\text{1}},\;Z_{j}\;\right)\neq \text{0}$, then the estimates of the model obtained by ordinary least squares (OLS) are likely to be biased.

In contrast, the hybrid method [20] employed in this paper is motivated by the hierarchical structure underlying the fixed-effect (FE) and random effects (RE) models. It is based on the principle that the total effect of any variable $X_{ij}$ at the individual level (level 1) is comprised of two components, the between-cluster ${\overset{―}{X}}_{j}$ and within-cluster components ${(X}_{ij}-{\overset{―}{X}}_{j})$ that can have different effects on an outcome variable. The specification of the hybrid model allows for separate within- and between-cluster effects for level 1 and level 2 variables:


$$Y_{ij}=\beta_{\text{0}}+\beta_{W}{(X}_{ij}-{\overset{―}{X}}_{j})+\delta Z_{j}+\beta_{B}{\overset{―}{X}}_{j}+\left(\eta_{j}+e_{ij}\right)\;$$ (4)

where $\eta_{j}$ is a community level error term, $\beta_{W}$ is the “within-cluster” effect and $\beta_{B}$ the “between-cluster” effect of $X_{ij}$. The within-cluster effect $\beta_{W}$ assesses how on average a within-cluster change in $X_{ij}$ is associated with a within-cluster change in outcome $Y_{ij}$ and since it is estimated using only within-cluster variation it is identical to the estimate obtained based on the fixed-effect method applied at the cluster level [25, 21]. The between-cluster effect $\beta_{B}$, assesses how a unit change in the average of X between clusters, i.e., ${\overset{―}{X}}_{j}$, is associated with a change in the average value of $Y_{ij}$ between clusters, i.e., ${\overset{―}{Y}}_{j}$.

The hybrid model in equation (4) is estimated as a standard random intercept model. A key assumption of the random intercept model is that residuals are independent of the covariates, i.e.,

$E\left(\eta_{j}|X_{ij},Z_{j}\right)=\;E\left(e_{ij}|X_{ij},Z_{j}\right)=\text{0}$. Specifically, the assumption $E\left(\eta_{j}|X_{ij},Z_{j}\right)=\text{0}$ requires that there be no omitted community-level confounders, or no unobserved heterogeneity at the cluster level. This assumption may be considered as more defensible given that the cluster mean values of all the covariates ${\overset{―}{X}}_{j}$, and not just the cluster mean of the policy variable of interest, are already included in the regression. Nevertheless, if the cluster-level error term is correlated with the covariates, i.e., $E\left(\eta_{j}|X_{ij},Z_{j}\right)\neq \text{0}$, then the effect estimates of a RE model will be biased. In contrast, the FE estimator does not require any assumption on the distribution of $\eta_{j}\;$, since it relies exclusively on variation within clusters.

The correlated random-effects model (CRE) [22], sometimes called the Mundlak model [21], is mathematically equivalent to the hybrid model in (4). The main difference is that it includes the level one variable $X_{ij}$ directly into the regression equation instead of the demeaned form used in the hybrid model (i.e., ${(X}_{ij}-{\overset{―}{X}}_{j}))$:


$$Y_{ij}=\beta_{\text{0}}+\beta_{\text{1}}X_{ij}+\delta Z_{j}+\left(u_{j}+e_{ij}\right)\;$$ (5)

In contrast to the standard random intercept model, the CRE model relaxes the assumption of zero correlation between the level two error, $u_{j}$, and the level 1 variables. Specifically, it assumes that $u_{j}$ depends on the cluster mean values of all the level-1 covariates, i.e.,


$$u_{j}=\gamma {\overset{―}{X}}_{j}+\eta_{j},$$ (6)

Substituting $u_{j}$ in equation (5) by $\gamma {\overset{―}{X}}_{j}+\eta_{j}$ yields

$Y_{ij}=\beta_{\text{0}}+\beta_{\text{1}}X_{ij}+\delta Z_{j}+\gamma {\overset{―}{X}}_{j}+\left(\eta_{j}+e_{ij}\right),\text{or}\;$


$$Y_{ij}=\beta_{\text{0}}+\beta_{W}X_{ij}+\delta Z_{j}+\gamma {\overset{―}{X}}_{j}+\left(\eta_{j}+e_{ij}\right)$$ (7)

where $\eta_{j}\sim N(\text{0},\sigma_{\eta_{j}}^{\text{2}})$. In equation (7), $\beta_{W}$ is an estimate of the “within-cluster” effect, given that including the mean of a level one variable ${\overset{―}{X}}_{j}$ in a random effects model is an alternative to cluster mean centering [26], and γ is the “contextual” effect that explicitly models the difference between the “between” and “within” effects, i.e.,. $\gamma =\beta_{B}-\beta_{W}$ and


$$Y_{ij}=\beta_{\text{0}}+\beta_{W}X_{ij}+\delta Z_{j}+\left(\beta_{B}-\beta_{W}\right){\overset{―}{X}}_{j}+\left(\eta_{j}+e_{ij}\right).$$ (8)

Equation (8) can be estimated using the random effects method and has the advantage that it is not subject to cluster-level unobserved heterogeneity and thus able to yield unbiased estimates of the variables varying within clusters as well as of those variables that do not vary within clusters but vary only between clusters, such as $Z_{j}$ and ${\overset{―}{X}}_{j}$.

The hybrid/CRE approach outlined above offers two main advantages. First, it provides a fresh and clear interpretation of the estimates of the coefficients of the individual and community level variables in the literature. The studies on externalities published to date rely on the standard OLS approach discussed above without any explicit recognition of the differences in the variation of variables or their effects within-clusters and between-clusters or the potential role of unobserved heterogeneity at the community level in biasing OLS estimates. Second, the framework provides the opportunity to assess the sensitivity of the estimates of externalities on the outcomes of interest to different assumptions regarding which or how many community level variables to include in the analysis of externalities. The majority of the studies on externalities focus on one policy variable of interest generated by averaging out individual level (level 1) data at the cluster level (level 2). Such variables are usually the years of education of a mother or access to improved sanitation. The assumption of the hybrid/CRE model that $u_{j}$ depends on the cluster mean values of all the level-one covariates, instead of just the one policy variable of interest, allows for externalities arising from other covariates at the cluster level and provides the opportunity to examine how this potential synergy could affect the estimate of the externality associated with the policy variable of interest. In the results section of our paper, we also investigate whether the estimate of the externality associated with a policy variable of interest, such as maternal education, is higher or lower when the community average of the fraction of households with improved toilets and with improved water and/or other community level covariates are also included in the model, in comparison to the externality estimate associated with the policy variable of interest when community level covariates are excluded from the regression.

An additional advantage is the ability to test the equality of within and between cluster effects, that can also be used as an alternative to the Hausman specification test [27]. If between and within effects are the same, i.e.,$\;\gamma =\beta_{B}-\beta_{W}=\text{0}$ then (8) collapses to the simple random-intercept model (3). A purely empirically-driven approach to the CRE model could be to assume that $u_{j}$ depends on the community average of all the level one variables in the model, test the random effects hypothesis for each level one variable, and then re-estimate the CRE model including the community level means of the variables for which the test rejects the RE hypothesis. The hybrid modeling approach can also be extended to include random slopes allowing the effects of child-level variables to vary between clusters.

One important issue associated with the construction of community-level (level 2) variables by averaging out individual-level (level one) variables in the community/cluster is the number of household observations in a cluster. A small number of households/individuals sampled in any given cluster gives rise to errors in the variable, a problem that leads to attenuation bias, and thus, towards an inappropriate failure to reject the null hypothesis (that is, towards finding that community effects are not important, when, in fact, they are). This problem is exacerbated by the practice of using non-self means derived by summing the variable of interest over the sample cluster and then subtracting the observation for the household and dividing this difference by the number of households in the cluster. Schunk explores the consequences of small cluster size in linear multilevel models of level 2 variables (such as cluster means) that are functions of level one variables [28]. Using simulations, he reports that small cluster sizes can cause severe downward bias in estimated regression coefficients of aggregated level two variables and that the bias does not decrease if the number of clusters (i.e., level 2 units) increases. In our analysis we calculate community level means based on the sample of households with children under 5 years of age because the hybrid modeling approach is applicable only to the sample being analyzed. Studies following the standard ad-hoc approach include in the community mean observations from households in the sampled cluster that do not have children below five years of age. (e.g., see [8]). Ultimately, the extent to which the use of non-self-means or the use of all households sampled in the cluster has any substantial impact on the estimates of the size of the estimated externalities is an empirical question.

The preceding discussion has argued that estimates of externalities based on OLS and ignoring synergies may be biased due to cluster-level unobserved heterogeneity that may be correlated with the included regressors. The next section examines the size of this bias at the empirical level and addresses the question of whether such bias leads to misleading conclusions about the role externalities at the community level. We have also explored the option of analyzing possible externalities associated with having simultaneous access to improved water and sanitation at the household level. Unfortunately, the sample sizes were not sufficient to support such an analysis.

## Results

Tables 3– report the estimates of externalities associated with OLS and hybrid models on the incidence of fever and diarrhea based on a linear probability model (LPM) in urban and rural areas respectively. We have also estimated logit models and confirmed that the main results were identical regarding the presence of externalities. In addition to the three policy variable of interests, the control variables used in the analysis included: the child’s age in months (7 categories), the child’s gender, whether the child is mother’s first child, whether mother’s height is more than 160 cm, whether the house has a high quality roof, total number of household members, total number of children under 5 in the household, travel time to urban center with at least 20,000 people, household asset index (5 quintiles) at the county level, binary variables for the year of survey, and binary variables for the month of survey.

**Table 3:** Effects of household-level and community/cluster level variables on child fever in urban areas, children 0 to 60 months.

VARIABLES	(1)	(2)	(3)	(4)	(5)	(6)
	OLS	OLS	OLS	Hybrid-3	Hybrid-All	CRE-All
**Household level/ Within-cluster effects**						
Mother’s Education (in yrs)	−0.000	0.002***	−0.000	0.0015***	0.0012**	0.0012**
	[−0.461]	[3.549]	[−0.461]	[3.43]	[2.83]	[2.83]
Household has improved water	−0.000	0.000	−0.000	−0.0032	−0.0042	−0.0042
(1 = Y, 0 = N)	[−0.111]	[0.085]	[−0.073]	[−0.70]	[−0.92]	[−0.92]
Household has improved, non-shared toilet	−0.011***	−0.014***	−0.014***	−0.0141***	−0.0146***	−0.0146***
(1 = Y, 0 = N)	[−2.804]	[−3.843]	[−3.659]	[−3.65]	[−3.73]	[−3.73]
**Cluster-level/Between-cluster effects**						
Mean Mother Education (in yrs)		−0.005***		−0.0040***	−0.0022**	−0.0034***
		[−5.660]		[−5.69]	[−2.72]	[−3.76]
Fraction of households in cluster with improved water			−0.001	0.0043	0.0146*	0.0189*
			[−0.060]	[0.58]	[1.88]	[2.06]
Fraction of households in cluster with improved toilet	−0.008			−0.0177**	−0.0179**	−0.0033
	[−1.046]			[−2.67]	[−2.36]	[−0.38]
Observations	80,856	80,856	80,856			
R squared	0.024	0.025	0.024			
N (Level 1)				80,856	80,856	80,856
N (Level 2)				6,951	6,951	6,951

**Source:** Author calculations based on DHS surveys listed in Table 1.

**Notes:** Additional control variables included: Child age in months (7 categories), child born in lean season, child is a girl, child is first child, mother’s height is more than 160 cm, the house has a high quality roof, total number of household members, number of children under 5, travel time to urban center with at least 20,000 people, household asset index (5 categories), binary variables for year of survey, and binary variables for month of survey. [t-values] are based on standard errors corrected for intra-cluster correlation.

* p < .05; ** p < .01; *** p < .001.

**Table 4:** Effects of household-level and community-level variables on child fever in rural areas, children 0 to 60 months.

VARIABLES	(1)	(2)	(3)	(4)	(5)	(6)
	OLS	OLS	OLS	Hybrid-3	Hybrid-All	CRE-All
**Household level/ Within-cluster effects**						
Mother’s Education (in yrs)	0.001***	−0.000	−0.000	0.0010**	0.0009**	0.0009**
	[2.725]	[−0.127]	[−0.075]	[2.62]	[2.29]	[2.29]
Household has improved water	−0.006**	−0.002	−0.005*	−0.0021	−0.0025	−0.0025
(1 = Y, 0 = N)	[−2.062]	[−0.636]	[−1.898]	[−0.73]	[−0.86]	[−0.86]
Household has improved, non-shared toilet	−0.014***	−0.014***	−0.006*	−0.0064**	−0.0076**	−0.0076**
(1 = Y, 0 = N)	[−4.440]	[−4.370]	[−1.728]	[−2.01]	[−2.36]	[−2.36]
**Cluster-level/Between-cluster effects**						
Mean Mother Education (in yrs)	−0.002***			−0.0022***	−0.0011*	−0.0020**
	[−3.338]			[−3.75]	[−1.70]	[−2.63]
Fraction of households in cluster with improved water		−0.008		−0.0078	−0.0018	0.0006
		[−1.533]		[−1.69]	[−0.38]	[0.11]
Fraction of households in cluster with improved toilet			−0.024***	−0.0305***	−0.0229***	−0.0153*
			[−3.319]	[−4.75]	[−3.45]	[−2.06]
Observations	183,086	183,086	183,086			
R squared	0.029	0.029	0.029			
N (Level 1)				183,086	183,086	183,086
N (Level 2)				11,994	11,994	11,994

**Source:** Author calculations based on DHS surveys listed in Table 1.

**Notes:** Additional control variables included: Child age in months (7 categories), child is a girl, child is first child, mother’s height is more than 160 cm, the house has a high quality roof, total number of household members, number of children under 5, travel time to urban center with at least 20,000 people, household asset index (5 categories), binary variables for year of survey, and binary variables for month of survey. [t-values] are based on standard errors corrected for intra-cluster correlation.

* p < .05; ** p < .01; *** p < .001.

**Table 5:** Effects of household-level and community-level variables on child diarrhea in urban areas, children 0 to 60 months.

VARIABLES	(1)	(2)	(3)	(4)	(5)	(6)
	OLS	OLS	OLS	Hybrid-3	Hybrid-All	CRE-All
**Household level/ Within-cluster effects**						
Mother’s Education (in yrs)	−0.001***	−0.003***	−0.003***	−0.0010***	−0.0010***	−0.0010***
	[−3.211]	[−9.109]	[−9.098]	[−2.78]	[−2.75]	[−2.75]
Household has improved water	0.001	−0.004	−0.000	−0.0045	−0.0046	−0.0046
(1 = Y, 0 = N)	[0.205]	[−1.098]	[−0.091]	[−1.13]	[−1.14]	[−1.14]
Household has improved, non-shared toilet	−0.014***	−0.013***	−0.016***	−0.0186***	−0.0178***	−0.0178***
(1 = Y, 0 = N)	[−4.479]	[−4.211]	[−4.678]	[−5.41]	[−5.14]	[−5.14]
**Cluster-level/Between-cluster effects**						
Mean Mother Education (in yrs)	−0.005***			−0.0060***	−0.0057***	−0.0046***
	[−7.235]			[10.31]	[−8.72]	[−6.13]
Fraction of households in cluster with improved water		0.010		0.0116*	0.0103	0.0148*
		[1.398]		[1.90]	[1.62]	[1.99]
Fraction of households in cluster with improved toilet			0.007	−0.0098	−0.0128*	0.0050
			[1.066]	[−1.72]	[−2.06]	[0.71]
Observations	80,897	80,897	80,897			
R squared	0.045	0.044	0.044			
N (Level 1)				80,897	80,897	80,897
N (Level 2)				6,951	6,951	6,951

**Source:** Author calculations based on DHS surveys listed in Table 1.

**Notes:** Additional control variables included: Child age in months (7 categories), child is a girl, child is first child, mother’s height is more than 160 cm, the house has a high quality roof, total number of household members, number of children under 5, travel time to urban center with at least 20,000 people, household asset index (5 categories), binary variables for year of survey, and binary variables for month of survey.[t-values] based on standard errors corrected for intra-cluster correlation.

* p < .05; ** p < .01; *** p < .001.

**Table 6:** Effects of household-level and community-level variables on child diarrhea in rural areas, children 0 to 60 months.

VARIABLES	(1)	(2)	(3)	(4)	(5)	(6)
	OLS	OLS	OLS	Hybrid-3	Hybrid-All	CRE-All
**Household level/ Within-cluster effects**						
Mother’s Education (in yrs)	0.001**	−0.002***	−0.002***	0.0006	0.0004	0.0004
	[2.078]	[−7.533]	[−7.531]	[1.65]	[1.03]	[1.03]
Household has improved water	−0.004**	−0.001	−0.005**	−0.0029	−0.0039	−0.0039
(1 = Y, 0 = N)	[−1.969]	[−0.510]	[−2.025]	[−1.14]	[−1.53]	[−1.53]
Household has improved, non-shared toilet	−0.006**	−0.006**	−0.004	−0.0057**	−0.0072***	−0.0072***
(1 = Y, 0 = N)	[−2.361]	[−2.203]	[−1.435]	[−2.03]	[−2.52]	[−2.52]
**Cluster-level/Between-cluster effects**						
Mean Mother Education (in yrs)	−0.006***			−0.0058***	−0.0043***	−0.0046***
	[−10.869]			[−13.25]	[−8.69]	[−7.71]
Fraction of households in cluster with improved water		−0.008*		−0.0059	0.0002	0.0041
		[−1.648]		[−1.64]	[0.05]	[0.90]]
Fraction of households in cluster with improved toilet			−0.006	−0.0101*	−0.0037	0.0035
			[−0.985]	[−1.97]	[−0.69]	[0.58]
Observations	183,100	183,100	183,100			
R squared	0.042	0.041	0.041			
N (Level 1)				183,100	183,100	183,100
N (Level 2)				11,993	11,993	11,993

**Source:** Author calculations based on DHS surveys listed in Table 1.

**Notes:** Additional control variables included: Child age in months (7 categories), child is a girl, child is first child, mother’s height is more than 160 cm, the house has a high quality roof, total number of household members, number of children under 5, travel time to urban center with at least 20,000 people, household asset index (5 categories), binary variables for year of survey, and binary variables for month of survey. [t-values] are based on standard errors corrected for intra-cluster correlation.

* p < .05; ** p < .01; *** p < .001.

In each of these tables, columns (1) to (3) present the estimates of the external effects obtained using the typical OLS approach used in the literature, whereby potential externalities are investigated by including the cluster level average of just one of the policy variables of interest, such as the average years of education of mothers in the cluster or the fraction of households in the cluster with improved water or improved toilets. Such a specification ignores the potential of any synergies at the cluster level among policy variables of other household observed characteristics. To the extent that the policy variable of interest such as higher maternal education at the community-level interacts with other community level variable such as the fraction of households with access to improved water or improved sanitation that are omitted from the regression model, then the estimate of the external effect of maternal education is likely to be biased.

In column (4) of Tables 3 through 8, the specification Hybrid-3 includes only the three policy variables of interest by restricting potential synergies with any other household variables at the cluster level. In column (5) the specification Hybrid-All is less restrictive since it takes into consideration that there may be synergies not only among the three policy variables but also other individual observed characteristics at the cluster level (level 1). The likelihood ratio tests comparing the estimates of the restricted model Hybrid-3 (using the cluster average of only the three policy variables of interest) with the unrestricted model Hybrid-All (using the cluster average of all the control variables at the individual level) indicated that the Hybrid-All model fitted the data better in all cases.

In sum, three main findings emerge from the preferred estimates in columns (5) and (6) of Tables 3 through 6 regarding the role of externalities on the incidence of fever and diarrhea. First, the Hybrid-All estimates in column (5) of Tables 3-6 reveal that there are significant externalities associated with maternal education at the community level with respect to the incidence of fever and diarrhea. Ceteris paribus, clusters with a higher average in years of education of mothers are significantly associated with a lower probability of incidence of child fever and diarrhea in both urban and rural areas. The faction of households with improved toilets is also significantly associated with a lower probability of incidence of child fever in both urban and rural areas and a lower probability of incidence of diarrhea in urban areas.

Second, a comparison of the estimates with the standard OLS model and the Hybrid-All model (col 5) reveals that externality estimates based on the standard OLS approach may be quite misleading. Depending on the outcome and the context, when synergies are considered for the effect size of community level externalities is lower. For example, the between-cluster coefficient of maternal education on child fever in urban areas is significantly lower than that obtained using just the cluster mean of maternal education (compare col 2 with col 5 in Tables 3 and 4). Thus, the traditional approach to estimating the presence of external effects is likely to result in an overestimate of the effect of the external effects.

Third, there are instances where within-cluster effect of maternal education on the reported incidence of child fever is opposite in sign than the between-cluster effect. For example, the within-cluster effect of maternal education on child fever in urban and rural areas (col 5 in Tables 3 and 4) is positive, whereas the between-cluster effect is negative. This suggests that ceteris paribus, mothers with more education may be more likely to report their children having fever, but at the cluster level, a higher average education is associated with a lower incidence of child fever.

In Tables 7 and 8 we present the respective estimates using child HAZ as an outcome. In contrast to the incidence of fever and diarrhea, that provide short-term measures of child health, child height for age (HAZ) is a measure of long-term child health and development. As such child HAZ may be better able to reflect the effects of synergies between maternal education at the community level and the fraction of households with improved water and/ or improved toilets.

**Table 7:** Effects of household-level and community-level variables on child HAZ in urban areas, children 0 to 60 months.

VARIABLES	(1)	(2)	(3)	(4)	(5)	(6)
	OLS	OLS	OLS	Hybrid-3	Hybrid-All	CRE-All
**Household level/ Within-cluster effects**						
Mother’s Education (in yrs)	0.022***	0.034***	0.034***	0.0222***	0.0219***	0.0219***
	[11.370]	[18.802]	[18.774]	[11.61]	[11.30]	[11.30]
Household has improved water	0.017	0.001	0.020	0.0094	0.0084	0.0084
(1 = Y, 0 = N)	[0.894]	[0.044]	[1.072]	[0.46]	[0.41]	[0.41]
Household has improved, non-shared toilet	0.121***	0.118***	0.065***	0.0937***	0.1011***	0.1011***
(1 = Y, 0 = N)	[7.310]	[7.025]	[3.919]	[5.62]	[5.99]	[5.99]
**Cluster-level/Between-cluster effects**						
Mean Mother Education (in yrs)	0.030***			0.0445***	0.0501***	0.0282***
	[8.736]			[15.21]	[15.27]	[7.42]
Fraction of households in cluster with improved water		0.049		0.0129	0.0141	0.0057
		[1.330]		[0.43]	[0.45]	[0.15]
Fraction of households in cluster with improved toilet			0.146***	0.2493***	0.1805***	0.0794*
			[4.289]	[8.40]	[5.59]	[2.20]
Observations	71,656	71,656	71,656			
R squared	0.116	0.114	0.115			
N (Level 1)				71,656	71,656	71,656
N (Level 2)				6,800	6,800	6,800

**Source:** Author calculations based on DHS surveys listed in Table 1.

**Notes:** Additional control variables included: Child age in months (7 categories), child is a girl, child is first child, mother’s height is more than 160 cm, the house has a high quality roof, total number of household members, number of children under 5, travel time to urban center with at least 20,000 people, household asset index (5 categories), binary variables for year of survey, and binary variables for month of survey. [t-values] are based on standard errors corrected for intra-cluster correlation.

* p < .05; ** p < .01; *** p < .001.

**Table 8:** Effects of household-level and community-level variables on child haz in rural areas, children 0 to 60 months.

VARIABLES	(1)	(2)	(3)	(4)	(5)	(6)
	OLS	OLS	OLS	Hybrid-3	Hybrid-All	CRE-All
**Household level/ Within-cluster effects**						
Mother’s Education (in yrs)	0.010***	0.027***	0.027***	0.0108***	0.0110***	0.0110***
	[6.356]	[17.733]	[17.828]	[6.65]	[6.68]	[6.68]
Household has improved water	0.051***	−0.030**	0.055***	−0.0259*	−0.0272**	−0.0272**
(1 = Y, 0 = N)	[4.487]	[−2.433]	[4.787]	[−2.13]	[−2.22]	[−2.22]
Household has improved, non-shared toilet	−0.009	−0.012	0.010	0.0438**	0.0521***	0.0521***
(1 = Y, 0 = N)	[−0.618]	[−0.834]	[0.666]	[3.06]	[3.61]	[3.61]
**Cluster-level/Between-cluster effects**						
Mean Mother Education (in yrs)	0.033***			0.0389***	0.0411***	0.0301***
	[11.879]			[16.97]	[16.00]	[9.86]
Fraction of households in cluster with improved water		0.174***		0.1180***	0.0854***	0.1126***
		[7.707]		[6.42]	[4.60]	[5.08]
Fraction of households in cluster with improved toilet			−0.054*	−0.0333	−0.0723**	−0.1245***
			[−1.704]	[1.27]	[−2.73]	[−4.15]
Observations	162,824	162,824	162,824			
R squared	0.118	0.117	0.116			
N (Level 1)				162,824	162,824	162,824
N (Level 2)				11,790	11,790	11,790

**Source:** Author calculations based on DHS surveys listed in Table 1.

**Notes:** Additional control variables included: Child age in months (7 categories), child is a girl, child is first child, mother’s height is more than 160 cm, the house has a high quality roof, total number of household members, number of children under 5, travel time to urban center with at least 20,000 people, household asset index (5 categories), binary variables for year of survey, and binary variables for month of survey. [t-values] are based on standard errors corrected for intra-cluster correlation.

* p < .05; ** p < .01; *** p < .001.

Maternal education at the household level as well as at the cluster level has a strong positive effect on child HAZ. In both urban and rural areas, accounting for synergies among the three policy variables and other household characteristics policy reveal externality effects associated with maternal education that are greater than those obtained from the standard OLS approach (compare estimates in col. 1–3 in Table 7 and 8 with those col. 5). The Hybrid-All estimates reveal that there are also substantial externalities at the community level associated with the fraction of households having access to improved toilets (Table 7, col. 5) in urban areas and with improved water in rural areas (Table 8, col. 5). Thus, OLS-based studies of externalities using the community average of the policy variable of interest alone, are likely to result in misleading estimates of the effects of other level one variables on the outcome of interest. This problem does not arise with the CRE approach whereby the within-cluster estimates of level one variables are independent of how many community level variables are included in the regression.

However, it is important to point out one associated with the estimates of improved water and improved toilets in rural areas. Estimates in rural areas seem to be affected by collinearity between the within and between variation. Such indications of collinearity arise from the reversal of the sign of the coefficient of improved water when the fraction of households in the cluster with improved water is included in the regression (the coefficient of 0.051 in Table 8 col 1, changes to −0.030 in Table 8 col 2). A similar pattern emerges for the improved toilets variable (the coefficient of −0.009 in Table 8 col 1, changes to 0.010 in Table 8 col 3). Bearing these important caveats in mind, in rural areas, access to improved toilets is associated with a positive effect on child height within clusters, but there appear to be no externalities arising from a higher fraction of households with improved toilets between clusters. The opposite pattern emerges for access to improved water. Ceteris paribus, access to improved water at the household level within communities is associated with lower child HAZ, but a higher fraction of households with access to improved water in the cluster is associated with a higher average child HAZ.

Table 9 provides a qualitative summary of the estimates of the between-cluster estimates (or externality effects of maternal education, improved water and improved sanitation on the three outcome variables analyzed in this paper.

**Table 9:** Summary of between-cluster (community level) effects by policy variable, and child health outcome for urban and rural areas in SSA.

	Fever	Fever	Diarrhea	Diarrhea	HAZ	HAZ
	Urban	Rural	Urban	Rural	Urban	Rural
**Maternal Education**						
Between-cluster effect	–	–	–	–	+	+
**Improved Water**						
Between-cluster effect	0	0	0	0	0	+/c
**Improved Toilets**						
Between-cluster effect	–	–	–	0	+	-/c

Notes: 0 Indicates coefficient that is not statistically different from 0; c Indicates the possible presence of collinearity.

The analysis so far has considered only a linear specification for externalities. In Table 10 we relax this assumption and use a quadratic specification for the cluster means of all the level 1 variables based on the hybrid model summarized in equation (4). The likelihood ratio tests comparing the estimates of the restricted model Hybrid-3 (using the quadratic specification for the cluster average of only the three policy variables of interest) with the unrestricted model Hybrid-All (using the quadratic specification of the cluster average of all the control variables at the individual level) indicated that the quadratic Hybrid-All model fitted the data better in all cases.

**Table 10:** Nonlinearities in effects of community-level variables.

VARIABLES	(1)	(2)	(3)	(4)	(5)	(6)
	Fever-U	Fever-R	Diarrhea-U	Diarrhea-R	HAZ-U	HAZ-R
Mean Mother Education (in yrs)	0.0056*	0.0056**	0.0015	0.0020	−0.0013	0.0393***
Mean Mother Education (in yrs) squared	−0.0005***	−0.0008***	−0.0005***	−0.0007***	0.0037***	0.0002
Fraction of households in cluster with improved water	0.0116	0.0035	0.0300	0.0081	−0.0804	0.0255
Fraction of households in cluster with improved water squared	0.0016	−0.0050	−0.0170	−0.0085	0.0834	0.0593
Fraction of households in cluster with improved toilet	0.0084	−0.0618***	0.0108	−0.0176	0.1429	−0.1001
Fraction of households in cluster with improved toilet squared	−0.0354	0.0524*	−0.0282	0.0190	0.0090	0.0439

**Source:** Author calculations based on DHS surveys listed in Table 1.

**Notes:** Additional control variables included: Mother’s education, household having access to improved water, household having access to improved toilet, child age in months (7 categories), child is a girl, child is first child, mother’s height is more than 160 cm, the house has a high quality roof, total number of household members, number of children under 5, travel time to urban center with at least 20,000 people, household asset index (5 categories), binary variables for year of survey, and binary variables for month of survey. Standard errors corrected for intra-cluster correlation in brackets.

* p < .05; ** p < .01; *** p < .001.

The estimates reveal that the quadratic term for maternal education at the community level is statistically significant in most instances such as for child height-for-age in urban areas, and for fever and diarrhea in rural areas. Also, the coefficients of improved toilets for fever in rural areas (Table 10, col 2) also suggest that there are increasing returns associated with a higher fraction of households with improved toilets at the community level. The current estimates suggest that the threshold value beyond which decreasing returns set in (or the marginal returns of improved sanitation equal zero), is when 59% of the households in the community have improved toilets. Given that this is almost three times the current mean fraction of households with improved toilets in rural areas (about (20%; see Table 2) this implies that positive externalities associated with improved toilets in rural areas will continue to be at work for a while in the foreseeable future.

### Concluding remarks and policy considerations

In this paper we used data from twenty countries from the harmonized versions of the DHS surveys for countries in Sub-Saharan Africa to investigate the extent to which contextual community factors impact the nutrition and health of children less than 60 months of age. We used the level of women’s schooling in the community and the fraction of households in the community with improved water and the fraction with improved sanitation facilities as the community factors. Long-term child health was measured by child height for age z-scores, and short-term child health by the incidence of reported fever and diarrhea in the two weeks preceding the survey.

We employed the hybrid modeling approach that combines the strengths of random-and fixed-effects models. First, it allows for the accounting of unobserved heterogeneity at the community level which has been largely ignored in the literature up to now. Second, it also allows for the inclusion of all the potential synergies in externalities at the community level. Third, it permits testing the sensitivity of the estimates of externalities to alternative specifications of the cluster-level unobserved heterogeneity.

The empirical analysis confirms the role of externalities at the community level and shows that the size of these externalities varies between rural and urban areas and by outcome considered. The preferred hybrid approach reaffirmed the paramount role of externalities in maternal education on child height-for-age (in both urban and rural areas), in the incidence of fever (in urban areas) and the incidence of diarrhea (in both urban and rural areas). Significant externalities also appear to be associated with the fraction of households in the community having improved toilets reducing fever (in both urban and rural areas) and diarrhea in urban areas as well as in increasing child HAZ.

An important advantage of the hybrid model is that it also yields estimates of the within-cluster effects of the same policy variables. These effects are independent of the potential influence of unobserved heterogeneity at the community level. Again, both mother’s education and improved toilets have significant effects within clusters.

Our findings inform public policy decisions on the amounts of public investments in education and basic water and sanitation infrastructure, whether costs of such investments need to be subsidized as part of broader development strategy, and whether public resources should be targeted to the community as opposed to the household level. Solely from the lens of child health outcomes (both in terms of nutrition and the incidence of illness) investments in improved water do not appear to improve HAZ or reduce the incidence of fever or diarrhea in our sample of children less than 60 months of age. However, given data limitations, the measure of improved water may not indicate safe water, especially if the improved water is non-piped [29]. But, increasing the years of education of mothers and increasing household-specific access to improved toilets through public policy will have not only private returns to the family with more educated mothers and improved infrastructure but also benefits to other children in the community. The clearest case where public funding provides benefits outside of private returns to the beneficiary of the policy is in education. Additional investments in having educated mothers not only accrues health benefits to the mother’s own children but also to the other children in the community. While changing the educational attainment of current mothers is more challenging, investing in the education of girls, future mothers, is an investment in the health of all the children in their community.
